# A Collection of Pre-mRNA Splicing Mutants in *Arabidopsis thaliana*

**DOI:** 10.1534/g3.119.400998

**Published:** 2020-04-07

**Authors:** Tatsuo Kanno, Peter Venhuizen, Ming-Tsung Wu, Phebe Chiou, Chia-Liang Chang, Maria Kalyna, Antonius J. M. Matzke, Marjori Matzke

**Affiliations:** * Institute of Plant and Microbial Biology, Academia Sinica, 128, Sec. 2, Academia Rd., Nangang District, Taipei, 11529 Taiwan; † Department of Applied Genetics and Cell Biology, University of Natural Resources and Life Sciences - BOKU, Muthgasse 18, 1190 Vienna, Austria; ‡ Department of Plant Sciences, University of Cambridge, Downing Street, CB2 3EA Cambridge, UK

**Keywords:** *Arabidopsis thaliana*, CBP80, miRNAs, mutant screen, pre-mRNA splicing

## Abstract

To investigate factors influencing pre-mRNA splicing in plants, we conducted a forward genetic screen using an alternatively-spliced *GFP* reporter gene in *Arabidopsis thaliana*. This effort generated a collection of sixteen mutants impaired in various splicing-related proteins, many of which had not been recovered in any prior genetic screen or implicated in splicing in plants. The factors are predicted to act at different steps of the spliceosomal cycle, snRNP biogenesis pathway, transcription, and mRNA transport. We have described eleven of the mutants in recent publications. Here we present the final five mutants, which are defective, respectively, in RNA-BINDING PROTEIN 45D (*rbp45d*), DIGEORGE SYNDROME CRITICAL REGION 14 (*dgcr14*), CYCLIN-DEPENDENT KINASE G2 (*cdkg2*), INTERACTS WITH SPT6 (*iws1*) and CAP BINDING PROTEIN 80 (*cbp80*). We provide RNA-sequencing data and analyses of differential gene expression and alternative splicing patterns for the *cbp80* mutant and for several previously published mutants, including *smfa* and new alleles of *cwc16a*, for which such information was not yet available. Sequencing of small RNAs from the *cbp80* mutant highlighted the necessity of wild-type CBP80 for processing of microRNA (miRNA) precursors into mature miRNAs. Redundancy tests of paralogs encoding several of the splicing factors revealed their functional non-equivalence in the *GFP* reporter gene system. We discuss the cumulative findings and their implications for the regulation of pre-mRNA splicing efficiency and alternative splicing in plants. The mutant collection provides a unique resource for further studies on a coherent set of splicing factors and their roles in gene expression, alternative splicing and plant development.

Splicing of pre-mRNAs by the excision of introns and ligation of flanking exons is a prerequisite for the expression of most eukaryotic genes. Splicing entails two transesterification reactions carried out by the spliceosome, a large and dynamic ribonucleoprotein (RNP) machine located in the nucleus. At least six structurally and functionally distinct spliceosomal complexes containing core spliceosomal proteins, transiently-associated factors and different combinations of five different small nuclear (sn) RNAs - U1, U2, U4, U5 and U6 – act sequentially to execute the two catalytic steps of the splicing process (Matera and Wang 2014; Yan *et al.*, 2017). The spliceosome is able to carry out constitutive splicing, in which the same splice sites are always used for a given intron, and alternative splicing, in which splice site usage for a given intron is variable. Alternative splicing increases transcriptome and proteome diversity (Nilsen and Graveley 2010; Syed *et al.*, 2012; Reddy *et al.*, 2013) and is important for development and stress adaptation in plants (Staiger and Brown 2013; Filichkin *et al.*, 2015; Szakonyi and Duque 2018).

Most information on spliceosome composition and the splicing mechanism has been derived from genetic, biochemical and structural studies in yeasts and metazoan cells (Papasaikas and Vacárcel 2016). Structural and mechanistic insights into the splicing process in these organisms have relied heavily on the development of manipulable biochemical systems that perform the splicing reactions *in vitro*. Similar work has lagged in plants, largely owing to the lack of an efficient *in vitro* splicing system (although see recent progress in this area; Albaqami and Reddy 2018). Many of the approximately 430 predicted splicing-related proteins encoded in the *Arabidopsis thaliana* (Arabidopsis) genome have been identified through sequence similarity searches with yeast, *Drosophila* and human genes (Koncz *et al.*, 2012). Genetic approaches have been useful for revealing the physiological roles for some of these splicing factors. For example, forward genetic screens designed to investigate requirements for distinct processes, such as hormone responses (Hugouvieux *et al.*, 2001; Zhan *et al.*, 2015) and flowering time (Marquardt *et al.*, 2014), have identified different splicing-related proteins. Reverse genetics has been used to study the consequences of specific splicing factor deficiencies at particular developmental stages (Ali *et al.*, 2007; Szakonyi and Duque 2018) or under different environmental conditions (for example, Zhan *et al.*, 2015; Laloum *et al.*, 2018; Calixto *et al.*, 2018. Cavallari *et al.* 2018; Huertas *et al.*, 2019). However, to our knowledge, an unbiased forward genetic screen dedicated to identifying factors that influence alternative splicing of a well-defined alternatively spliced gene has not previously been conducted in any plant system.

We developed a novel *GFP* reporter system in Arabidopsis to carry out such a forward genetic screen. In this system, an intron-containing *GFP* reporter gene present in a wild-type ‘Target’ (WT T) line exhibits variable levels of *GFP* expression depending on the splicing pattern of its pre-mRNA. Of three major *GFP* splice variants present in wild-type plants, only one, which arises from splicing a U2-type intron with comparatively weak, non-canonical AU-AC splice sites, produces a translatable *GFP* mRNA. The other two *GFP* transcripts – a spliced transcript resulting from excision of a U2-type intron with strong, canonical GU-AG splice sites, and an unspliced pre-mRNA – cannot be translated because they comprise premature termination codons. Our working hypothesis is that mutations in genes encoding splicing factors will alter the relative proportions of the three splice variants, leading to an increase or decrease in the level of translatable *GFP* mRNA. Theoretically, these changes should result, respectively, in either a ‘Hyper-GFP’ (HGF) or ‘GFP-weak’ (GFW) phenotype compared to the WT T line, which displays an intermediate level of GFP fluorescence (Kanno *et al.*, 2016; 2017a, b; 2018a,b).

Using the *GFP* splicing reporter system, we conducted a classical forward genetic screen to identify mutants exhibiting altered splicing and *GFP* expression. By screening for changes in GFP fluorescence in seedlings derived from chemically mutagenized seed, we retrieved nine *hgf* mutants and seven *gfw* mutants. Eleven of the mutants, which indeed turned out to be defective in various splicing-related factors, have been published previously (Sasaki *et al.*, 2015; Kanno *et al.*, 2016; 2017a,b; 2018a,b). Here we report the identity of the final five mutants together with previously unpublished information on global gene expression and alternative splicing profiles in one new mutant and in several mutants for which this information has not been previously published. Our study provides foundational knowledge for further in depth investigations of the splicing factors retrieved in the screen, and allows an integrated analysis of a set of sixteen splicing-related proteins unified by their involvement in processing the same splicing reporter.

## Materials and Methods

### Plant materials

All wild-type and mutant plants used in this study are in the ecotype Col-0 background and were cultivated under long-day conditions (22-23°, 16 hr light, 8 hr dark).

The T-DNA insertion mutants used in this study were as follows: SAIL_569_G05 (*rbp45a*; AT5G54900), SAIL_505_E03 (*rbp45b*; AT1G11650), SALK_063484C (*rbp45c*; AT4G27000), SALK_009736C, SAIL_527_G04, SALK_132471, SALK_053475 and SALK_152624 (*cwc16b*; AT1G17130), SAIL_608_B05 (*smfb*; AT2G14285). All the seeds were obtained from the Nottingham Arabidopsis Stock Center (NASC).

### Forward genetic screen

**Figure 1:**
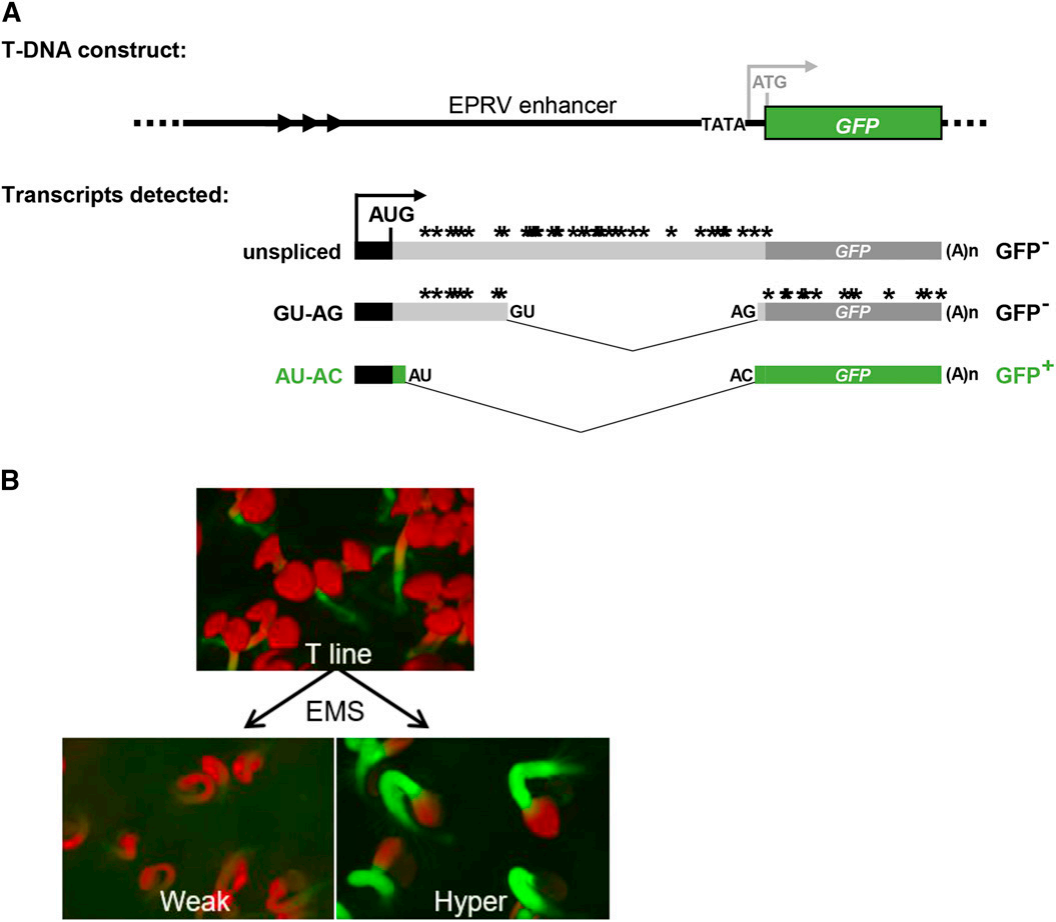
Alternatively-spliced GFP reporter gene system. (A) The T-DNA construct introduced into Arabidopsis comprises a *GFP* reporter gene under the transcriptional control of a minimal promoter (TATA) and upstream viral (EPRV) enhancer. In the WT T line, however, the expected transcription initiation site (TSS) (light gray arrow) is not used. Rather transcription of *GFP* pre-mRNA initiates at an unanticipated upstream TSS (black bar and arrow). Alternative splicing yields three *GFP* splice variants: an unspliced transcript, a transcript resulting from splicing of a canonical GU-AG intron, and a transcript arising from splicing a U2-type intron with non-canonical AU-AC splice sites, which are considered weak compared to GU-AG splice sites (Crotti *et al.*, 2007). The unspliced and GU-AG transcripts contain numerous premature termination codons (black asterisks). Hence only the AU-AC transcript can be translated into GFP protein. The coding sequence of GFP protein (green bars) uniquely contains a 27 amino acid extension (short green bars with black outline) compared to standard GFP (Fu *et al.*, 2015; Kanno *et al.*, 2016). Arrowheads denote a tandem repeat cluster upstream of the cryptic promoter. The black AUG designates the major translation initiation codon. The 3′ AC splice site is only three nucleotides downstream of the 3′ AG splice site (Kanno *et al.* 2008; 2016, 2017a, b). (B) Schematic of forward screen and GFP fluorescence phenotypes of newly germinated seedlings. The wild-type Target (T) line has an intermediate level of GFP fluorescence visible primarily in the stem (hypocotyl) and shoot and root apices. Mutants generated by EMS treatment of the T line exhibit either reduced (Weak) or enhanced (Hyper) GFP fluorescence relative to the T line. Cotyledons (first leaves appearing from a germinating seedling) appear red owing to auto-fluorescence of chlorophyll at the excitation wavelength for GFP. Figure adapted from Figure 1 in Kanno *et al.* (2018a) with permission from the Genetics Society of America.

The forward genetic screen based on an alternatively-spliced, intron-containing *GFP* reporter gene in Arabidopsis [T line; referred to herein as ‘wild-type’ (WT)] (Figure 1A) has been described in detail in prior publications (Sasaki *et al.*, 2015; Kanno *et al.*, 2016; 2017a, b; 2018a,b). Briefly, approximately 40,000 Arabidopsis seeds of the WT T line homozygous for the alternatively-spliced *GFP* reporter gene were treated with ethyl methane sulfonate (EMS) and sown on soil (M1 generation). From approximately 30,000 M1 plants that grew to maturity and produced self-fertilized seeds, 52 batches of M2 seeds (the first generation when a recessive mutation can be homozygous) were harvested. Surface-sterilized M2 seeds were germinated on solid Murashige and Skoog (MS) medium in plastic Petri dishes and examined under a fluorescence stereo microscope at seven days post-germination for GFP fluorescence. M2 seedlings showing enhanced or reduced GFP fluorescence relative to the WT T line were placed into *hgf* and *gfw* categories, respectively, and selected for further analysis (Figure 1B). Causal mutations were identified in the mutants by next generation mapping (NGM; James *et al.* 2013) using pooled DNA isolated from at least 50 BC1F2 progeny displaying the desired GFP phenotype. BC1F2 plants were produced by backcrossing the M2 plants with the WT T line followed by self-fertilization of the resulting BC1 progeny. Screening approximately 210,000 M2 seedlings (representing around seven M2 seedlings for each M1 plant) yielded nine *hgf* mutants and seven *gfw* mutants (Table 1). Mutations were confirmed by identification of multiple alleles and/or complementation analyses. All mutations reported here are recessive.

**Table 1:** Mutants identified in a forward genetic screen based on an alternatively-spliced *GFP* reporter gene

*Hyper-GFP (hgf)* mutant	Name	AGI number	Predicted function in splicing	No. of alleles	Effect of mutation on development	Reference
*hgf1*	coilin	At1g13030	marker protein for Cajal bodies, which facilitate snRNP maturation	12*^a^*	negligible	Kanno *et al.*, 2016
*hgf2*	CWC16a	At1g25682	step I factor	3*^a^*	negligible	Kanno *et al.*, 2017a
*hgf3*	SMU1	At1g73720	recruited prior to B* complex formation;	1	negligible	Kanno *et al.*, 2017a
*hgf4*	SMFA	At4g30220	small nuclear ribonucleoprotein	1	negligible	Kanno *et al.*, 2017a
*hgf5*	PRP39A	At1g04080	U1 snRNP component	5*^a^*	negligible	Kanno *et al.*, 2017b
*hgf6*	RBP45D	At5g19350	U1 snRNP component	2	negligible	this study
*hgf7*	DG CR14-related	At3g07790	spliceosomal C complex	2	negligible	this study
*hgf8*	CDKG2	At1g67580	splicing-related protein kinase	1	early flowering	this study
*hgf9*	IWS1	At1g32130	transcription elongation	2	negligible	this study
*gfw1*	AtRTF2	At5g58020	contributes to ubiquitin-based regulation of the spliceosome?	2	embryo lethal	Sasaki *et al.*, 2015; Kanno *et al.*, 2017a
*gfw2*	PRP8A	At1g80070	U5 snRNP component; acts at catalytic core of spliceosome	3	embryo lethal	Sasaki *et al.*, 2015; Kanno *et al.*, 2017a
*gfw3*	RBM25	At1g60200	U1 snRNP component	2	low seed set	Kanno *et al.*, 2017b
*gfw4*	PRP18A	At1g03140	step II factor	1	short roots, small siliques	Kanno *et al.*, 2018a
*gfw5*	PRP4KA	A3g25840	recruited prior to B* complex formation; needed for catalytic activation of spliceosome	5	broad rosettes, late flowering, tall stature, low seed set	Kanno *et al.*, 2018b
*gfw6*	SAC3A	At2g39340	mRNA export factor	5	negligible	Kanno *et al.*, 2018b
*gfw7*	CBP80	At2g13540	multiple	1	Serrated leaves, early flowering	this study
T line (WT)	n.a.	n.a.	Wild-type line expressing *GFP* reporter gene; used for EMS mutagenesis	n.a.	normal	

The mutants retrieved in a forward genetic screen based on an alternatively-spliced *GFP* reporter gene in Arabidopsis (Figure 1) include a predicted core spliceosomal protein (SMFa); putative components of the U1 snRNP (PRP39a, RBM25, RBP45d) and U5 snRNP (PRP8); putative step I and step II factors transiently associated with the spliceosome (CWC16a and PRP18a, respectively); a predicted complex C protein (DGCR14); putative splicing regulatory proteins (RTF2, SMU1, PRP4ka, CDKG2); one structural protein presumed to be important for snRNP maturation (coilin), putative mRNA export factors (SAC3a, CBP80) and a predicted transcription elongation factor (IWS1). Developmental phenotypes are primarily observed in six (of seven) identified *gfw* mutations, two of which are embryo-lethal.

a Further screening of the M2 population after publication of the first alleles of coilin, *PRP39a* and *CWC16a* has identified three new alleles of coilin (R9H; first intron, 3′ splice site; second intron, 5′ splice site), one new *prp39a* allele (R226*) and two new *cwc16a* alleles (W18*; fifth intron, 3′ splice site). These unpublished alleles are counted in the number of alleles shown here.

Abbreviation: SRA, Sequence Read Archive (NCBI); ABRC, Arabidopsis Biological Resource Center; T (or ST) refers to the WT T line harboring the alternatively-spliced *GFP* reporter gene. If the sequencing data from T line has a separate SRA number, it is noted in the table; n.d., not done; n.a., not available.

### Complementation

For complementation of the *rbp45d-1*, *dgcr14-1*, *cdkg2-3*, *iws1-2* and *cbp80-1* mutants, the respective coding sequences (https://www.arabidopsis.org/) were modified to add *Sal*I sites and *Xba*I at the 5′ and 3′ ends, respectively, and to replace internal *Sal*I or *Xba*I sites by silent mutations. The modified cDNAs were synthesized by Genscript (www.genscript.com) and cloned into pUC57 as *Sal*I-*Xba*I fragments.

The binary vector BV-Mp*PAT*ot *Sal*I (Matzke *et al.*, 2010) (PAT, phosphinothricin acetyltransferase, which confers resistance to DL-phosphinothricin) was altered to contain the 35S promoter from cauliflower mosaic virus (35Spro) (Pietrzak *et al.*, 1986) and 3C transcriptional terminator region (from the pea *rbcS3C* gene (Benfey *et al.*, 1990) between the PAT selection marker and the left T-DNA border region of the binary vector. Between the 35Spro and 3C terminator region, a chloramphenicol resistance marker was inserted between the *Sal*I site after the 35Spro and the *Xba*I site positioned before the 3C terminator. By cutting with *Sal*I and *Xba*I, the chloramphenicol resistance marker could be replaced by the modified CDS (under transcriptional control of the 35Spro) of genes to be complemented.

To permit immunoprecipitation of IWS1 in future experiments, the *IWS1* cDNA (lacking a stop codon) was fused to three copies of a C-terminal FLAG tag. For this, an adaptor consisting of a *Spe*I fragment encoding three copies of a FLAG peptide sequence with a stop codon followed by *Xba*I NPTII *Xba*I *Spe*I fragment (NPTII, neomycin phosphotransferase conferring resistance to kanamycin) was synthesized by Genscript. This adaptor was inserted in the correct orientation into the *XbaI* site of the modified binary vector that contains an *IWS1* cDNA lacking a stop codon, by selecting for kanamycin resistance in bacteria. The *NPTII* selection marker was deleted afterward with *Xba*I, thus reconstituting the 35Spro-*IWS1-FLAG* CDS.

Binary vectors containing the modified cDNAs were introduced into *Agrobacterium tumefaciens* strain ASE (Wu *et al.*, 2018) via tri-parental mating (Matzke and Matzke,1986) and Arabidopsis plants were transformed using the floral dip procedure (Clough and Bent 1998).

### RNA-sequencing and whole genome resequencing

Total RNA was isolated from approximately 80 mg of two week-old seedlings (BC1F3 generation) of each mutant line tested in this study (EMS-generated mutants: *cbp80*, *smfa-1*, *cwc16a-1*, *cwc16a-2*, *cwc16a-3*; SALK T-DNA insertion line: *smfb*) and wild type T plants using a Plant Total RNA Miniprep kit (GMbiolab, Taiwan) according to the manufacture’s protocol for the Lysis Solution B, which contains SDS/anti-oxidant. Briefly, the seedlings were ground into a fine powder in liquid nitrogen, then lysed in the Lysis Solution B for 10 min at 60°, followed by column purification steps. The purified total RNA was extracted using nuclease-free water after the on-column DNase treatment. Construction of libraries and RNA-seq were carried out (biological triplicates for each sample) as described previously (Sasaki *et al.* 2015; Kanno *et al.* 2016).

Whole genome re-sequencing of the EMS-generated mutants *cbp80*, *smfa-1*, *cwc16a-1*, *cwc16a-2*, *cwc1-a-3* was performed to identify any remaining EMS-induced second-site mutations that change splice sites. Alternative splicing events containing mutations were excluded from further analysis. To prepare the sample for whole genome re-sequencing, genomic DNA was isolated by DNeasy plant mini kit (Qiagen, Taiwan) followed by concentrating by Genomic DNA Clean & Concentrator (Zymo research, CA, USA) according to respective manufacturer’s instructions. Briefly, about 100 mg of two-week-old seedlings were used as starting material and the purified DNA was eluted from the column by 200 μL of the elution buffer (provided by the kit). The DNA solution was then concentrated to 60 μL via column concentration steps. Construction of libraries and DNA-seq were carried out as described previously (Sasaki *et al.*, 2015; Kanno *et al.*, 2016).

### Analysis of RNA-seq data for differential gene expression and differential alternative splicing

#### Differential expression analysis:

To determine differential expression of the *cbp80*, *coilin*, *cwc16a*, and *smfa* and *smfb* mutants compared to their respective WT T controls, we estimated the transcript per million (TPM) expression with Salmon (version 0.13.1; Patro *et al.*, 2017) for the Reference Transcript Dataset for *Arabidopsis thaliana* 2 (AtRTD2)-Quantification of Alternatively Spliced Isoforms (QUASI) (AtRTD2-QUASI) annotation (Zhang *et al.* 2017). Transcript read counts were grouped per gene using tximport (Soneson *et al.* 2015) and differentially expressed genes were determined using edgeR with the exactTest (version 3.18.1; Robinson *et al.* 2010). Genes were considered differentially expressed for a false discovery rate (FDR) < 0.05.

#### Read alignment:

Reads were mapped to the index based on the TAIR10 genome release (Lamesch, *et al.* 2012) and the AtRTD2 transcriptome with STAR (version 2.6.0c; Dobin *et al.* 2013) using a 2-pass mapping. The following parameters were used:–outSAMprimaryFlag AllBestScore,–outFilterMismatchNmax 2/0 (first/second pass),–outSjfilterCountTotalMin 10 5 5 5,–outFilterIntronMotifs RemoveNoncanonical,–alignIntronMin 60,–alignIntronMax 6000, –outSAMtype BAM SortedByCoordinate. During the second pass, the splice junction files of the relevant control and test samples were passed to the mapping via the–sjdbFileChrStartEnd flag.

#### Alternative splicing analysis:

Alternative splicing events were obtained and quantified using Whippet (version 0.11; Sterne-Weiler *et al.* 2018). Two separate splice graph indices were generated; one for the detection and quantification of exon skipping (ES), alternative acceptor (AA) and alternative donor (AD) events, and another for the retained introns (RI) and exitron (EI) events. Exitrons are alternatively spliced internal regions of protein-coding exons (Marquez *et al.*, 2015). Both indices were based on the AtRTD2 transcriptome annotation (Zhang *et al.* 2017), supplemented with the relevant STAR RNA-seq alignments and were generated with the–bam-both-novel and–bam-min-reads 3 flags. The RI/EI index was further supplemented with ‘pre-mRNA’ coordinates of the genes and the exitron splice junctions detected using an in-house script. The ‘pre-mRNA’ coordinates range from the start to the end of the gene and allow us to quantify the retention levels of all annotated introns in a gene. The whippet delta step was run with default parameters, except for the–min-samples 3 flag. The alternative acceptor and donor events were filtered, assuring that at least both (alternative) junctions were detected in the Whippet data. The RI events were required to be covered by at least one read for either all control and/or test samples. All events with a probability ≥ 0.9 and an absolute delta percent-spliced-in (PSI) ≥ 0.1 were considered significant differential alternative splicing events.

#### SNP/indel calling:

SNPs and indels were identified using the Genome Analysis Toolkit (GATK) pipeline (Van der Auwera *et al.* 2013). Picard (version 2.10.9, http://broadinstitute.github.io/picard) was used to generate the sequence dictionary for the TAIR10 genome release. Reads were aligned to the TAIR10 genome using BWA-MEM (0.7.16a-r1181; Li 2013), with the added -M flag. The resulting SAM file was converted to BAM format, sorted, and duplicates were marked using Picard tools. The GATK (version 3.8-0-ge9d806836) HaplotypeCaller was used to obtain the raw variants and the SelectVariants function was used to extract the SNPs and indels. SNPs were filtered using the following filter expression: “QD < 2.0 || FS > 60.0 || MQ < 40.0 || MQRankSum < −12.5 || ReadPosRankSum < −8.0.” The filter expression for indels was as follows: “QD < 2.0 || FS > 200.0 || ReadPosRankSum < −20.0.”

SNPs and indels were intersected with the AtRTD2 annotated transcripts and the significantly regulated alternative splicing nodes from Whippet using in-house scripts.

#### RT-PCR to detect GFP splice variants:

Total RNA was isolated as described above but without the on-column DNase treatment. Twenty-five microliters of the RNA solution were then treated with two units of RQ1 RNase-Free DNase (Promega, USA) in a total reaction volume of 50 μl according to the manufacturer’s instructions. The DNase treated RNA was purified by NucleoSpin RNA Clean-up kit (Macherey-Nagel, Germany) and eluted with 60 μl of nuclease-free water. Following the manufacturer’s protocol, cDNA was made by Transcriptor First Strand cDNA Synthesis Kit (Roche, USA) from 1 μg of the purified RNA and an oligo d(T) primer as a template and a primer, respectively. RT-PCR was carried out under the following conditions: [94° for 2 min followed by 28 cycles of 94° for 10 s, 58° for 20 s, and 72° for 90 sec, and finally 72° for 7 min] or [94° for 2 min followed by 24 cycles of 94° for 10 s, 58° for 20 s, and 72° for 30 s, and finally 72° for 7 min] for detecting *GFP* transcripts or Actin transcripts, respectively. Primers are shown in Table S1.

### Detecting GFP protein by Western blotting

Approximately 100 mg of two-week-old seedlings grown on solid MS medium in plastic Petri dishes were frozen in liquid nitrogen, disrupted into a fine powder by TissueLyser II (Qiagen, USA) and resuspended in 100 µl of extraction buffer A (50 mM HEPES-KOH pH 7.9, 400 mM KCl, 2.5 mM MgCl2, 1 mM EDTA, 1 mM DTT, 0.1% Triton X-100) supplemented with EDTA-free protease inhibitor cocktail (Roche, USA). The suspension was vortexed three times for 15 sec and centrifuged for 10 min at maximum speed at 4°. The supernatants were mixed with equal volumes of extraction buffer A without KCl (sample A). The pellet was resuspended in 200 µl of extraction buffer B (50 mM HEPES-KOH pH 7.9, 200 mM KCl, 2.5 mM MgCl2, 1 mM EDTA, 1 mM DTT, 0.1% Triton X-100) supplemented with EDTA-free protease inhibitor cocktail (Roche, USA) and sonicated three times for eight seconds, 10% duty cycle and 20% power (Bandelin Sonoplus HD 2070 with MS 73 probe), followed by centrifugation for 10 min at maximum speed at 4°. The supernatants were mixed with sample A. Protein concentrations in the samples were measured using the Bradford assay. Five hundred nanograms of protein were separated by ExpressPlus PAGE Gel, 4–12% (Genescript, Taiwan), transferred to Amersham Hybond P 0.2 μm PVDF Membrane (GE Healthcare, USA), followed by Western blotting according to standard procedures. Rabbit anti-tubulin (AS10 680; Agrisera, Sweden), and mouse anti-GFP (CPA9022; Cohesion bioscience, Taiwan) antibodies were used at 1:1,000 dilutions. Secondary antibody, goat anti-rabbit IgG-conjugated with horseradish peroxidase (Agrisera, Sweden) and goat anti-mouse IgG-conjugated with horseradish peroxidase (Biorad, USA), were used, respectively, at a 1:10,000 dilution. The blots were developed using Amersham ECL Select Western Blotting Detection Reagent kit (GE Healthcare, USA).

### PCR to identify gene knock-outs of T-DNA insertion mutants

To isolate genomic DNA from the T-DNA insertion mutant lines, several two week-old seedlings were frozen in liquid nitrogen, disrupted into fine powder by TissueLyser II (Qiagen, USA) and resuspended in 250 µl of extraction buffer (100 mM Tris-HCl pH 8.0, 500 mM NaCl, 50 mM EDTA, 10mM β-mercaptoethanol). After adding 35 µl of 10% SDS, the samples were incubated for 10 min at 65°. After ammonium acetate precipitation and isopropanol precipitation followed by washing and drying steps, the DNA pellet was dissolved in an appropriate volume of TE buffer (10 mM Tris-HCl pH 8.0, 1 mM EDTA). Genotyping was carried out by PCR using specific primers listed in Table S1. PCR conditions were as follows [94° for 2 min followed by 35 cycles of 94° for 10 s, 58° for 20 s, and 72° for 1 min, and finally 72° for 7 min]. When genotyping by using CAPS or dCAPS methods, the PCR products were digested by a suitable restriction enzyme (Table S1).

### Small RNA analysis in the cbp80-1 mutant

Total RNA was isolated from two-week old seedlings of the WT T line and *cbp80-1* mutants using a PureLink Plant RNA Reagent (Thermo Fisher, USA) and MaxTract high-density gel tubes (Qiagen, USA) following the manufacturer’s instructions. The quality and quantity of isolated RNA were checked with Agilent Bioanalyzer prior to usage. RNA concentrations were assessed by NanoDrop (ND-1000 spectrophotometer). Ten micrograms total RNA of each mutant line were used for library preparation and sRNA sequencing (Illumina HiSeq 2500 system) by an in house Genomic Technology Core Facility.

After the quality and adaptor trimming, the clean sRNA tags were processed according to a previously published procedure (Wu *et al.* 2017). In brief, the sRNA tags of 18–26 nt were mapped to the Arabidopsis genome (Release TAIR10) (Lamesch, *et al.* 2012) with Bowtie (Langmead *et al.* 2009). Tags that exhibited more than 20 genomic hits or were mapped to chloroplast genome, mitochondria genome, rRNAs, tRNAs, snRNAs or snoRNAs were discarded. To carry out cross-library comparison, the read numbers are used with normalization in transcripts per fifty million (TP50M). The normalized read counts of tag sequences from individual sequencing libraries were calculated by dividing the raw value by the total abundance of adjusted total raw counts of each library, and then multiplied by 50 million.

Northern blotting of sRNA was performed as described previously (Lee *et al.*, 2015) with a few modifications. Briefly, ten micrograms of total RNA were separated on 10% denaturing polyacrylamide TBE-Urea gels (Thermo Fischer, USA) and transferred to Hybond-N+ membranes (GE Healthcare, USA) using Electro Blot Mini System (Major Science, Taiwan). The membrane was then UV cross-linked with 120-mJ energy and baked for 1 h at 80°. DNA oligonucleotides complementary to miRNA (miRBase version 21) (Kozomara *et al.*, 2019) were used as probes to determine the expression of miRNA (sequences of probes in legend of Table S6). The probes were γ-^32^P end-labeled using T4 polynucleotide kinase (New England Biolabs, USA). Probe hybridizations were performed using Ultrahyb-Oligo buffer (Thermo Fischer, USA) at 37° overnight. After washing with buffer containing 2× SSC and 0.1% SDS, the membranes were exposed on PhosphorImager screens, and scanned using the Typhoon Scanner (GE Healthcare, USA). These membranes were also exposed to X-ray film for 1-7 days.

### Data availability

Figure S1 contains data on the *rbp45d* mutants; Figure S2: contains data on the *dgcr14* mutants Figure S3 contains data on the *cdkg2* mutant; Figure S4 contains data on the *iws1* mutant; Figure S5 contains data on the cbp80 mutant; Figure S6 contains a Northern blot analysis of selected miRNAs in the *cbp80-1* mutant; Figure S7 shows data on a redundancy test of *SMFA* and *SMFB*; Figure S8 contains data on the *cwc16a* mutants; CWC16; Table S1 contains primer sequences; Table S2 contains comparative phenotypic data on the *cbp80-1* mutant, *cbp80* complemented plants and wild-type; Table S3 contains an analysis of differential alternative splicing events in the *cbp80* mutant; Table S4 contains an analysis of differentially expressed genes and miRNAs in the *cbp80* mutant; Table S5 contains an analysis of differential alternative splicing events in the coilin mutants; Table S6 contains an analysis of differentially expressed genes in the coilin mutants; Table S7 contains an analysis of differential alternative splicing events in the *cwc16a* mutants; Table S8 contains an analysis of differentially expressed genes in the *cwc16a* mutants;

Table S9 contains an analysis of differentially expressed genes in the *smfa* and *smfb* single mutants; Table S10 contains an analysis of differential alternative splicing events in the *smfa* and *smfb* single mutants; Table S11 contains an analysis of differential alternative splicing events in the *smfa smfb* double mutant; Table S12 contains an analysis of differentially expressed genes in the *smfa smfb* double mutant.

Seeds of all mutant and wild-type *Arabidopsis thaliana* lines listed in Table 1 are available at the Arabidopsis Biological Resource center (ABRC, Ohio, USA) and all DNA and RNA sequence data for selected mutants and the wild-type T line are available at NCBI under the respective accession numbers listed as follows. *hgf1*/coilin/At1g13030/ ABRC stock number: CS69632, CS69639; NCBI accession numbers: R40*/hgf1-1 and *P439L*/*hgf1-8*: SRP071829, T line: SAMN12817582, *P439L/hgf1-8:*SAMN12817583, R40*/hgf1-1: SAMN12817584, T line, *P439L/hgf1-8*, and R40*/hgf1-1: SRP089352 and SRP089656. *hgf2*/CWC16a/At125682/ ABRC stock number: CS69846, CS72366, CS72367; NCBI accession numbers: this study, *cwc16a-1*: SRP093582, T line: SAMN12817589, *cwc16a-1:* SAMN12817590, *cwc16a-2:* SAMN12817591, *cwc16a-3:* SAMN12817592. *hgf3*/SMU1/At1g73720/ ABRC stock number: N623852; NCBI accession numbers: *smu1-1*: SRP093582. *hgf4*/SMFA/At4g30220/ ABRC stock number: CS69848; NCBI accession numbers: T line: SAMN12817585, *smfa:* SAMN12817586, *smfb:* SAMN12817587, *smfab:* SAMN12817588. *hgf5*/PRP39A/At1g04080/ ABRC stock number: CS69936, CS69937, CS69640; NCBI accession numbers: *prp39a-3* and *prp39a-4*: SRP108084, T line: SRP093582. *hgf6*/RBP45D/At5g19350/ ABRC stock number: CS72358, CS72359; NCBI accession numbers not determined. *hgf7*/DG CR14- related/At3g07790/ ABRC stock number: CS72360, CS72361; NCBI accession numbers not determined. *hgf8*/CDKG2/At1g67580/ ABRC stock number: CS72362; NCBI accession numbers not determined. *hgf9*/IWS1/At1g32130/ ABRC stock number: CS72363, CS72364; NCBI accession numbers are not determined. *gfw1*/AtRTF2/At5g58020/ ABRC stock number: CS69596, N540515; NCBI accession numbers: T line: SRR1652313, *atrtf2-1*: SRR1652314, *atrtf2-2* heterozygous: SRR1652316, *atrtf2-2* homozygous: SRR1652317. *gfw2*/PRP8A/At1g80070/ ABRC stock number: CS69597; NCBI accession numbers: *prp8-7*: SRR1652315. *gfw3*/RBM25/At1g60200/ ABRC stock number: CS69940, CS69941; NCBI accession numbers not determined. *gfw4*/PRP18A/At1g03140/ ABRC stock number: CS69984; NCBI accession numbers: *prp18a-1*: SRP119240, T line: SRP093582 and SRP119240. *gfw5*/PRP4KA/A3g25840/ ABRC stock number: CS71818; NCBI accession numbers: *prp4ka-4*: SRP117313. *gfw6*/SAC3A/At2g39340/ ABRC stock number: CS71820; NCBI accession numbers: *sac3a-6*: SRP117313. *gfw7*/CBP80/At2g13540/ ABRC stock number: CS72365; NCBI accession numbers: T line: SAMN12817580, *cbp80:* SAMN12817581, T line and *cbp80*: SRP089656 and SRP089665. The T line (WT)/ ABRC stock number: CS69640 is the control for all the RNA-seq experiments. Supplemental material available at figshare: https://doi.org/10.25387/g3.11369361.

## Results

**Figure 2:**
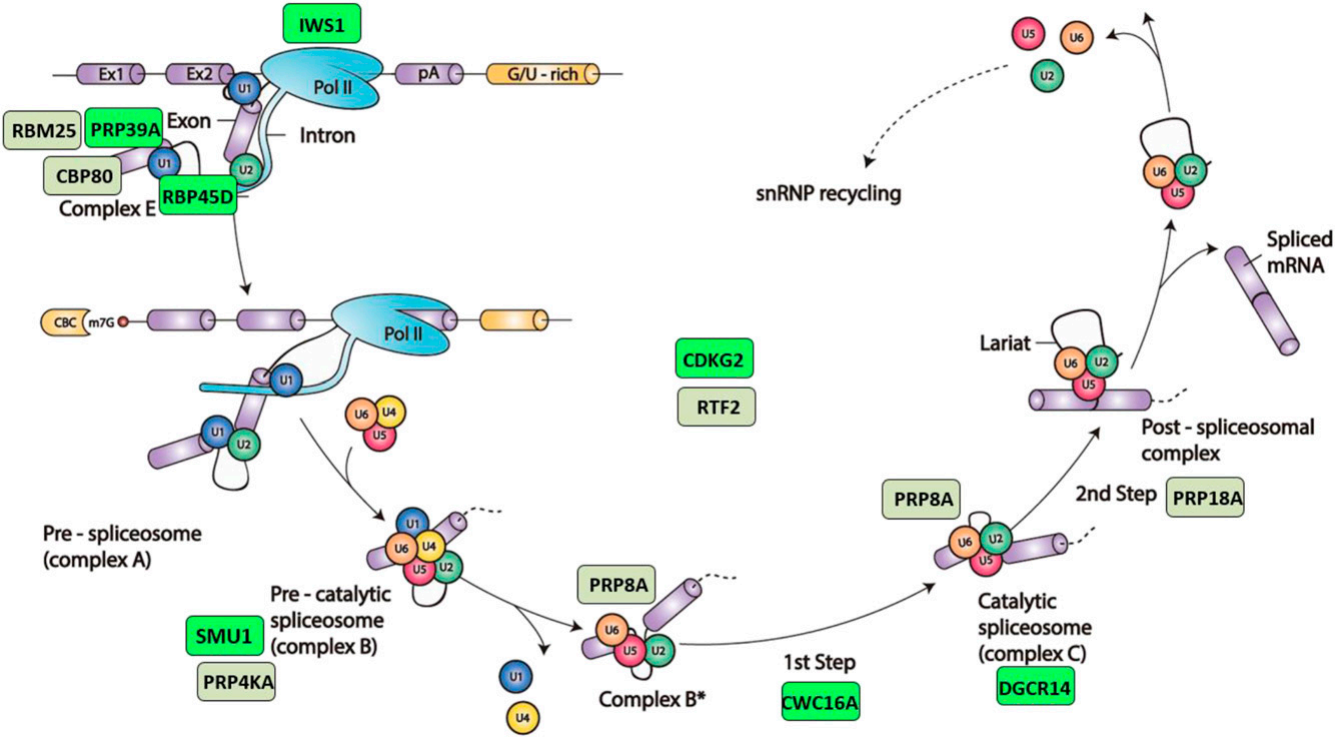
Spliceosomal cycle and factors identified in the forward genetic screen. Splicing is catalyzed by the spliceosome, a large and dynamic ribonucleoprotein (RNP) machine located in the nucleus. Spliceosomes comprise five small nuclear (sn) RNPs, each containing a heptameric ring of Sm or Like-Sm proteins and a different snRNA (U1, U2, U4, U5 or U6), as well as numerous other non-snRNP proteins. During the splicesomal reaction cycle, the five snRNPs act sequentially on the pre-mRNA with a changing assemblage of non-snRNP proteins to form a series of complexes that catalyze two consecutive *trans*-esterification reactions. In complex E, U1 and U2 snRNPs first recognize the 5′ and 3′ splice sites branch points of introns and interact to form pre-spliceosomal complex A. The subsequent addition of preformed U4/U5/U6 tri-snRNP creates pre-catalytic complex B. Ensuing reorganization steps induce release of U1 and U4 snRNPs and conversion of complex B to complex B*, which catalyzes the first reaction yielding the free 5′ exon and lariat 3′-exon intermediates. The newly formed C complex catalyzes the second reaction to achieve intron lariat excision and exon ligation. Lastly, dismantling of the spliceosome frees individual components to assemble anew at the next intron. The positions of factors identified in our screen, as predicted by their orthologs in yeast or metazoans, are indicated by colored rectangles (green, Hyper-GFP; dull green, GFP-weak). Adapted by permission from Springer Nature, Nat. Rev. Mol. Cell Biol. 15: 108–121, A day in the life of the spliceosome, A. G. Matera and Z. Wang, 2014.

**Figure 3:**
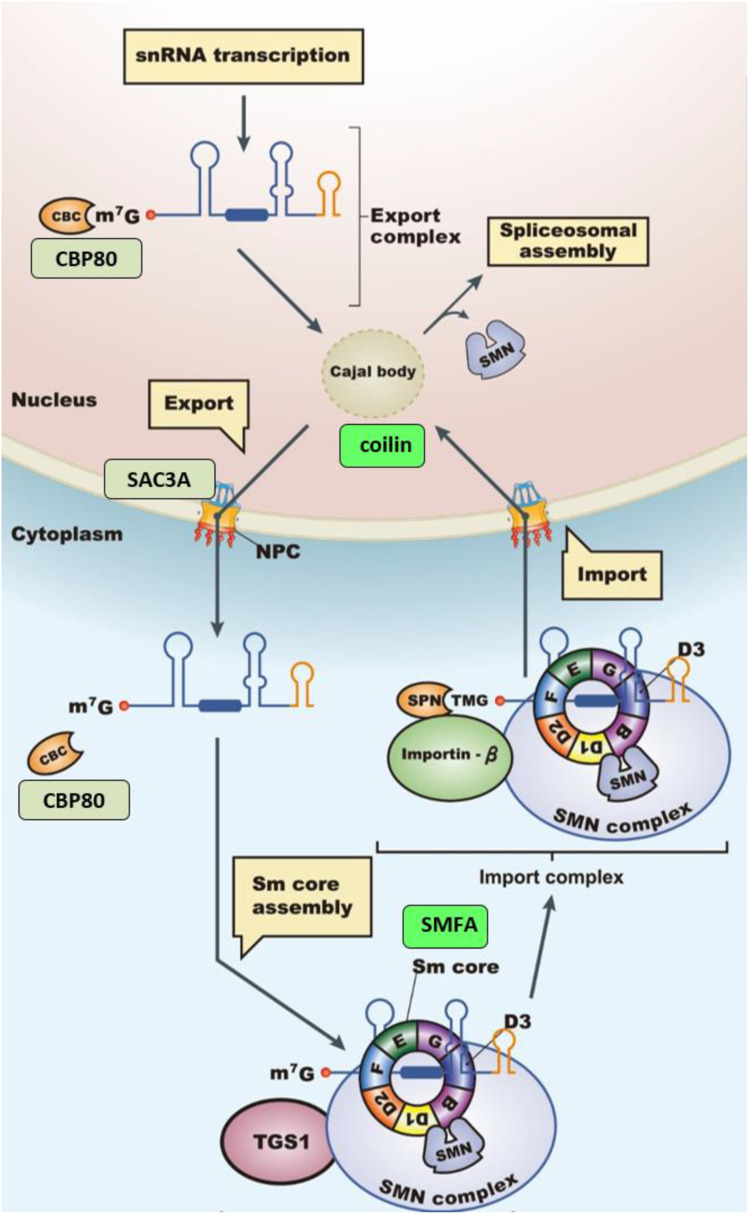
snRNP maturation pathway and factors identified in the forward genetic screen snRNAs are transcribed by RNA polymerase II in the nucleus and transported to the Cajal Body (CB), where they undergo quality control steps before export to the cytoplasm. In the cytoplasm, the monomethyl guanosine 5′ cap is converted to trimethyl guanosine by the enzyme TGS1. The snRNAs are encircled by a heteromeric ring complex comprising seven Sm core proteins, which protect the snRNA and together with the trimethyl guanosine cap, act as nuclear import signals. Back in the nucleus, the snRNP transits again through the CB where it undergoes further maturation steps before being released into the nucleoplasm to assemble into spliceosomes. Predicted positions of factors identified in our screen are shown by colored rectangles (bright green, Hyper-GFP; dull green, GFP-weak). Adapted by permission from Springer Nature, Nat. Rev. Mol. Cell Biol. 15: 108–121, A day in the life of the spliceosome, A. G. Matera and Z. Wang, 2014.

The sixteen factors identified in the forward genetic screen are listed in Table 1. All correspond to putative splicing-related proteins predicted to act at a number of steps of the spliceosomal cycle and snRNP biogenesis pathway (Figure 2 and Figure 3, respectively). More than one allele was retrieved for ten of the mutants, suggesting that the screen is close to saturation. Eleven of the mutants have been reported previously. The published *hgf* mutants include *coilin/hgf1* (Kanno *et al.*, 2016); *cwc16a/hgf2*, *smu1/hgf3*, *smfa/hgf4* (Kanno *et al.*, 2017a); and *prp39a/hgf5* (Kanno *et al.*, 2017b). The *gfw* category includes *rtf2/gfw1* and *prp8a/gfw2* (Kanno *et al.*, 2017a); *rbm25/gfw3* (Kanno *et al.*, 2017b); *prp18a/gfw4* (Kanno *et al.*, 2018b); and *prp4ka/gfw5* and *sac3a/gw6* (Kanno *et al.*, 2018b). Here we present the final five mutants: four in the *hgf* category (*rbp45d*, *dgcr14*, *cdkg2*, and *iws1*) and one in the *gfw* group (*cbp80*).

### New hgf mutants

#### rbp45d/hgf6 – At5g19350:

NGM analysis of two mutants placed in the *hfg6* group revealed mutations in the gene encoding RBP45D (RNA-Binding Protein 45D). Arabidopsis RBP45D, which is 425 amino acids in length, is a nuclear RNA binding protein that contains three RRM (RNA Recognition Motif) domains and preferentially binds to nuclear polyA^+^ RNA *in vitro* (Lorković *et al.*, 2000). The two *rbp45d* mutations that we recovered result, respectively, in a premature termination codon (W306*; *rbp45d-1*) and a frame shift caused by a mutation in the splice site acceptor of the 6^th^ (out of seven) intron (*rbp45d-2*). (Figure S1A). A wild-type copy of the *RBP45D* gene complemented the hyper-GFP phenotype when introduced into an *rbp45d* mutant (Figure S1B). The two *rbp45d* mutants did not show any obvious growth or developmental abnormalities.

The RBP45D orthologs in budding yeast and humans - Nam8p and TIA-1, respectively – are U1 snRNP components that stimulate splicing at weak splice sites (Gottschalk *et al.*, 1998; Förch *et al.*, 2000, 2002). Although RBP45D has been shown previously to lack this capability, an Arabidopsis protein similar to RPB45D, termed UBP1, appears to enhance splicing at otherwise inefficiently spliced introns (Lorković *et al.*, 2000). In the alternatively-spliced *GFP* reporter system, somewhat increased levels of both the translatable *GFP* AU-AC transcript, which may contribute to the hyper-GFP phenotype of the *rpb45d* mutant, and the non-translatable GU-AG transcript were observed (Figure S1C). These results might indicate that wild-type RBP45D can, in some cases, suppress splicing at both weak and strong splice sites. Another possibility is that mutations in RBP45D contribute to a hyper-GFP phenotype through a mechanism other than splicing regulation. Further work is needed to investigate these possibilities.

*RBP45D* has three paralogs in Arabidopsis: *RBP45A* (At5g54900), *RBP45B* (At1g11650) and *RBP45C* (At4g27000). All four *RBP45* genes are ubiquitously expressed, although *RBP45D* is expressed at a lower level than its three paralogs (http://bar.utoronto.ca/). We tested whether mutations in *RBP45A*, *RBP45B*, and *RBP45C* would affect expression of the *GFP* reporter gene by crossing the respective T-DNA insertion mutants with the WT T line. The F2 seedlings descending from these crosses were genotyped for homozygosity of the desired T-DNA insertion mutation. Unlike the *rbp45d* mutants, the *rbp45a*, *rbp45b* or *rbp45c* mutants, which represent T-DNA knockout insertions (Figure S1D), did not display a hyper-GFP phenotype or increased accumulation of GFP protein relative to the WT T line (Figure S1B and S1E, respectively). The *RBP45* paralogs are thus functionally nonequivalent in splicing in the *GFP* splicing reporter system.

#### dgcr14/hgf7 – At3g07790:

NGM analysis of two mutants placed in the *hfg7* group revealed mutations in the gene encoding DGCR14-like [DiGeorge Syndrome Critical Region, also termed ESS2 (Expression Studies2) in humans (Lindsay *et al.*, 1996) and EsS-2 (ES2-Similar) in *Caenorhabditis elegans* (Noma *et al.*, 2014)]. DiGeorge Syndrome is a human pleiotropic developmental disorder that is caused by a chromosome 22 deletion, the shortest of which contains the *DGCR14* gene. DGCR14-related proteins, which belong to the ESS2 superfamily of proteins, are evolutionarily conserved from fission yeast to humans. ESS2 proteins are typically around 500 amino acids in length and contain two predicted coiled-coil domains but no other recognizable functional domains.

There are not yet any published reports on DGCR14-like proteins in plants. In Arabidopsis, *DGCR14-like* is a single copy, intronless gene that encodes a protein 509 amino acids in length. We recovered two alleles in the screen: *dgcr14-1* (Q80*) and *dgcr14-2* (W365*) (Figure S2A). The corresponding mutants show a hyper-GFP phenotype and increased accumulation of GFP protein, which can be complemented with a wild-type copy of the *DGCR14-like* gene (Figure S2B and S2C). No obvious growth, morphological or reproductive defects were observed in the *dgcr14* mutants. *DGCR-like* is ubiquitously expressed in Arabidopsis (http://bar.utoronto.ca/) and predicted to encode a nuclear-localized protein (http://suba.live/).

From biochemical studies in human cells, DGCR14 appears to be most abundant in the spliceosomal C complex (Hegele *et al.*, 2012) (Figure 2). This affiliation remains to be confirmed in plants. The mechanism by which DGCR14 acts in splicing is not yet known. In *C. elegans*, ESS-2 has been found to foster accurate mRNA splicing when a splice site contains non-canonical sequences (Noma *et al.*, 2014). The increased level of the translatable AU-AC *GFP* transcript in *dgcr14* mutants suggests that the wild-type DGCR14 protein can suppress splicing at weak splice sites (Figure S2D), but additional experiments are required to confirm this idea.

#### cdkg2/hgf8 – At1g67580:

NGM analysis of the *hgf8* mutant identified a mutation in the gene encoding CDKG2 (Cyclin-Dependent Kinase G2). CDKs constitute an evolutionarily conserved group of serine/threonine kinases that have diverse roles in eukaryotes including cell cycle regulation, transcriptional modulation, pre-mRNA splicing, and translation (Doonan and Kitsios 2009). In humans CDKG11, which together with CDKG10 is most closely related to plant CDKGs, is particularly noted for its role in splicing (Doonan and Kitsios 2009; Cavallari *et al.*, 2018). Arabidopsis CDKG2 has not yet been implicated in mRNA processing; however, its close homolog, CDKG1 (At5g63370), was reported to associate with the spliceosome and regulate splicing of a gene involved in pollen wall formation (Huang *et al.*, 2013). CDKG1 was also found to modulate temperature-sensitive alternative splicing of a factor involved in regulation of flowering time in a pathway that involves CDKG2 in the thermo-sensing mechanism (Cavallari *et al.*, 2018).

CDKG2 is a ubiquitously expressed, nuclear protein that is 752 amino acids in length (http://bar.utoronto.ca/; http://suba.live/). Two T-DNA insertion alleles, *cdkg2-1* and *cdkg2-2* (SALK_012428 and SALK_090262) have been published previously (Ma *et al.*, 2015). The allele we isolated in the screen, *cdkg2-3*, results in a D530N substitution that alters a highly conserved amino acid in the highly conserved kinase domain (Figure S3A). The position of the *cdkg2-3* mutation suggests the kinase activity of CDKG2 is important for its function in splicing in the *GFP* reporter gene system but this remains to be tested in the future. The level of the translatable AU-AC *GFP* transcript increases substantially in the *cdkg2-3* mutant, which presumably accounts for the hyper-GFP phenotype of the mutant (Figure S3B). By contrast only trace amounts of the non-translatable GU-AG and unspliced transcripts can be detected by RT-PCR (Figure S3C). The *cdkg2-3* mutant has a normal appearance but it is somewhat early flowering (approximately four days before WT plants under our long-day growth conditions), as has been shown previously with the *cdkg2*-1 and *cdkg-2* T-DNA insertion alleles mentioned above (Ma *et al.*, 2015).

#### iws1/hgf9 – At1g32130:

The *hgf9-1* mutation was identified by NGM as an allele of the evolutionarily conserved transcription factor *IWS1* (Interacts With Spt6). In yeast, Iws1 has been shown to act in a complex with RNA polymerase II and Spt6 to facilitate pre-mRNA splicing, efficient mRNA export, and transcription elongation accompanied by histone H3K36 methylation, which is a mark of transcriptionally active chromatin (Yoh *et al.*, 2008).

The IWS1 protein in Arabidopsis, which is 502 amino acids in length, has a negligibly-expressed paralog, IWS2 (At4g19000) (Li *et al.*, 2010). Two alleles of *iws1*, *iws1-2* and *iws1-3*, were recovered in the screen. The *iws1-2* mutation alters the acceptor site of the 9^th^ intron, which is the penultimate intron, and the mutation in *iws1-3* results in a P446L amino acid substitution (Figure S4A). The hyper-GFP phenotype of *iws1-2* can be complemented by a wild-type copy of the *IWS1* gene (Figure S4B and S4C). The *iws1-2* mutant plants are viable and do not display any obvious defects in growth, development or reproduction under normal growth conditions. Mutations in *IWS1* were also isolated in independent forward screens in Arabidopsis for factors required for brassinosteroid-induced gene expression (*seb1*; *suppressor of bes1-d*) (Li *et al.*, 2010) and for High Nitrogen Insensitive (HNI) plants (Widiez *et al.*, 2011). IWS1 thus appears to participate in multiple physiological processes in plants, which probably reflects its predicted participation in transcription elongation, pre-mRNA splicing, epigenetic modulation, and mRNA export (Yoh *et al.*, 2008, Li *et al.*, 2010; Widiez *et al.*, 2011).

It is unclear how the *iws1* mutations we identified confer a hyper-GFP phenotype. Epigenetic modifications, including various histone marks, are known to affect splicing efficiency (Luco *et al.*, 2011; Naftelberg *et al.*, 2015; Godoy Herz and Kornblihtt 2019). Conceivably, IWS1-dependent histone modifications in the *GFP* transcribed region could be altered in the *iws1* mutants, thus potentially affecting splicing of *GFP* pre-mRNA. However, the splicing pattern of *GFP* pre-mRNA changes only slightly in the *iws1-2* and *iws1-3* mutants (Figure S4D). An alternative role for IWS1 in modulating transport of *GFP* mRNA from the nucleus to the cytoplasm is conceivable but remains to be further examined in the context of the hyper-GFP phenotype of the *iws1* mutants.

### New gfw mutant

#### cbp80/gfw7 – At2g13540:

NGM analysis of the *gfw7* mutant identified a mutation in the gene encoding *CBP80* (*Cap Binding Protein 80*). CBP80, together with CBP20, forms the heterodimeric cap binding complex (CBC), which binds the 7-methylguanosine cap at the 5′-end of eukaryotic mRNAs. The CBC participates in multiple processes in the cell, including transcription, splicing, transcript export, and translation (Figure 2 and Figure 3) (Kuhn *et al.*, 2008; Gonatopoulos-Pournatzis and Cowling 2014). The CBC was initially isolated biochemically from HeLa cells, where it was shown to be important for splicing (Izaurralde *et al.*, 1994). In Arabidopsis, the ortholog of CBP80 was first identified genetically in a forward screen for abscisic acid (ABA) hypersensitive (*abh1*) mutants (Hugouvieux *et al.*, 2001).

The Arabidopsis CBP80 protein is 848 amino acids in length. The mutation identified in this screen, *cbp80-1* (= *abh1-9*), creates a premature termination codon (W630*) in the middle of a conserved MIF4G (Middle domain of eIF4G), type II domain (Figure S5A). It is not clear which step(s) in *GFP* pre-mRNA processing and transport is affected in the *cbp80-1* mutant. A role in splicing is suggested by an increase in the untranslatable, unspliced *GFP* transcript and decrease in the translatable *GFP* transcript resulting from splicing at the AU-AC splice sites in the *cbp80-1* mutant (Figure S5B). However, one or more other CBP80-dependent steps important for *GFP* expression could also be affected (Figures 2 and 3). Further work in the future is needed to investigate this question.

In addition to its GFP-weak phenotype (Figure S5C), the *cbp80-1* mutant has a visible developmental phenotype featuring serrated rosette leaves, decreased rosette diameter, short stature and reduced seed set (Figure S5D, Table S2). These phenotypes, some of which have been noted previously in other *cbp80* mutants (Kuhn *et al.*, 2008; Montgomery and Carrington 2008), can be at least partially complemented by introducing a wild-type copy of the *CBP80* gene into the *cbp80-1* mutant, as can the GFP-weak phenotype (Figure S5C and S5D; Table S2).

To determine the effects of the *cbp80-1* mutation on global gene expression and alternative splicing patterns, we carried out triplicate RNA-seq and analyzed the sequencing data for differentially expressed genes (DEGs) and differential alternative splicing (DAS) events in the *cbp80-1* mutant. In agreement with earlier results obtained from another *cbp80* mutant using an RT-PCR alternative splicing panel (Raczynska *et al.*, 2010), we found that a number of transcripts are differentially spliced in the *cbp80-1* mutant (Table 2; Table S3). A notable feature of the DEG list in the *cbp80* mutant is the increased accumulation of 53 microRNA (miRNA) precursors (pri-miRNAs) (Table S4, sheet miRNA). The high representation of pri-miRNA-encoding genes in the list of up-regulated DEGs was reflected in the GO analysis, which identified highly significant increases in the expression of genes involved in gene silencing by miRNAs and posttranscriptional regulation (Table S4, GO_UP_P sheet). Subsequent sequencing of small RNAs from the *cbp80-1* mutant demonstrated that the heightened accumulation of pri-miRNA transcripts was paralleled by a decrease in many of the corresponding mature miRNAs (Figure S6). These results expand on prior findings from tiling array experiments, which demonstrated that CBP80 is required for pri-miRNA processing (Laubinger *et al.*, 2008; Kim *et al.*, 2008).

**Table 2:** Numbers of DEGs and DAS events in different splicing-related factors recovered in the forward genetic screen

Differentially Expressed Genes						
Direction of change		*cbp80*	*coilin* *^a^*	*cwc16a* *^a^*	*smfa*	*sfmb*
Up		2105	334	676	3121	1093
Down		567	435	265	1937	1299
Total Number of DEGs		2672	769	941	5058	2392
Supplemental Table		Table S4	Table S6	Table S8	Table S9	Table S9
**Differential Alternative Splicing**						
**Event**		***cbp80***	***coilin*** *^a^*	***cwc16a*** *^a^*	***smfa***	***sfmb***
AA		168 (8.1)	129 (44.9)	45 (14.2)	292 (13.4)	69 (19.5)
AD		199 (9.6)	17 (5.9)	27 (8.5)	244 (11.2)	37 (10.5)
CE		158 (7.7)	20 (7.0)	20 (6.3)	307 (14.1)	29 (8.2)
EI		83 (4.0)	13 (4.5)	26 (8.2)	158 (7.2)	37 (10.5)
RI		1456 (70.5)	108 (37.6)	200 (62.9)	1183 (54.2)	181 (51.3)
Total Number of DAS events		2064 (100)	287 (100)	318 (100)	2184 (100)	353 (100)
Total Number of DAS genes		1507	255	240	1465	287
Supplemental Table		Table S3	Table S5	Table S7	Table S10	Table S10

Abbreviations: AA, alternative acceptor/alternative 3′ splice site; AD, alternative donor/alternative 5′ splice site; CE, cassette exon; EI, exitron; RI, retained intron.

a Only the DEGs/DAS events changed in all alleles, with the same direction of change, are listed here. For the full overview of alternative splicing events per allele, see the respective supplementary tables.

### Redundancy tests

We previously carried out tests for functional redundancy of paralogs of *PRP39A*, *PRP18A* and *PRP4KA*, which were all identified in the *GFP* splicing reporter screen (Table 1). Similar to results obtained for *RBP45D* paralogs (see above), none of the paralogs tested previously (*PRP39B*, *PRP18B* and *PRP4KB*) were functionally equivalent to the A forms of the respective genes (Kanno *et al.*, 2017b, 2018a, b). In the present study, we tested *SMFB* for functional redundancy with its paralog, *SMFA*, which was identified in the screen as the *hgf4-1* mutant (P16*) (Table 1; Figure S7A and S7B). A knockout T-DNA insertion allele of *smfb* (Figure S7C) was introduced into the wild-type T line by crossing and F2 seedlings descending from the resulting F1 plants were screened for homozygous *smfb* progeny in a homozygous *TT* background. Unlike *smfa* homozygous seedlings, homozygous *smfb* seedlings displayed neither a hyper-GFP phenotype nor increased amounts of GFP protein (Figure S7B and S7C). Moreover, in contrast to *smfa*, which accumulated elevated levels of the translatable AU-AC *GFP* transcript relative to WT plants, the *GFP* pre-mRNA splicing pattern in the *smfb* mutant was virtually unchanged from the WT T line (Figure S7D). We thus conclude that *SMFA* and *SMFB* are functionally nonequivalent in the *GFP* reporter gene system.

A mutation in the gene encoding putative step 2 factor CWC16a (Figure 2) was initially identified in the GFP splicing reporter screen as *hgf2/cwc16a-1* (F50*) (Table 1) and two new alleles, *cwc16a-2* (W18*) and *cwc16a-3* (frame shift from amino acid 194), are reported here (Figure S8A). Attempts to test mutations in *CWC16B*, the paralog of *CWC16A*, were unsuccessful because of the five T-DNA insertion lines of *cwc16b* available from seed stock centers, three did not contain the T-DNA at the expected location and two did not appear to be *cwc16b* knockout alleles (Figure S8B). However, the fact that we recovered three alleles of *cwc16a* and none of *cwc16b* in our screen suggests a special role for CWC16A in splicing *GFP* pre-mRNA splicing. Indeed, the levels of the translatable AU-AC *GFP* transcript and the amount of GFP protein increase substantially in the *cwc16a* mutants (Figure S8C and S8D, respectively), which is fully consistent with their strong hyper-GFP phenotypes (Figure S8D). The lack of a convincing *cwc16b* knockout mutation prevented testing the splicing pattern of *GFP* pre-mRNA in a *cwc16b* mutant and the viability of a double *cwc16a cwc16b* mutant.

### Additional RNA-seq analyses from previously published mutants

We previously published RNA-seq data and analyses of DEGs and DAS events in two coilin mutants [(*hgf1-1* (R40*), *hgf1-8* (P439L)] and one allele of *cwc16a* (*cwc16a-1*). However, these earlier analyses lacked either an optimal number of biological replicates (coilin alleles; Kanno *et al.*, 2016) or were performed on only a single allele (*cwc16a-1*; Kanno *et al.*, 2017a). Here we expand on these prior results by reporting new RNA-seq data (in triplicate) and DEG and DAS analyses from the two coilin alleles mentioned above and from two newly identified alleles of *cwc16a*: *cwc16a-2* and *cwc16a-3* (Figure S8A-D). The more comprehensive results obtained by using multiple alleles of each mutant can be compared and lists of DEGs and DAS events shared by all alleles compiled. These comparisons hone the findings and provide a more accurate assessment of genes that robustly change in expression and alternative splicing profiles in a given mutant. The total number of shared DAS and DEG events among multiple alleles is shown in Table 2, with details, respectively, in Tables S5 and S6 (*coilin/hgf1* alleles) and Tables S7 and S8 (*cwc16a* alleles). A notable feature of the coilin DEG data are the highly significant representation of genes involved in metabolism of secondary compounds and responses to various stresses and environmental stimuli (Table S6, GO_share_UP_P). A previous albeit more limited transcriptome analysis of coilin mutants similarly suggested a prominent role for coilin in stress responses (Kanno *et al.*, 2016).

We also present here new triplicated RNA-seq data and DEG/DAS analyses for *smfa-1*, *smf-1b* and *smfa-1 smf-1b* double mutants. This information was not reported in a previous publication on *smfa-1* mutants (Kanno *et al.*, 2017a). An analysis of the RNA-seq data obtained with *smfa-1* or *smfb-1* single mutants revealed about twice as many DEGs (FDR < 0.05) in *smfa-1* compared to *smfb-1* (5058 and 2392, respectively, with 827 shared between the two paralogs) (Table 2; Table S9). More than 100 DEGs in *smfa-1*, compared to only seven in *smfb-1*, correspond to known or predicted splicing factors in Arabidopsis (numbering around 430; Koncz *et al.*, 2012) (Table S9_a_Koncz share and b_Koncz share). These results suggest that wild-type SMFA-1 is involved more frequently than SMFB-1 in modulating the expression of splicing-related factors. When considering DAS events, the difference between the two paralogs is even more pronounced (approximately sixfold). We identified 2184 cases of DAS for *smfa-1 vs.* 353 for *smf-1b*, with 98 instances of DAS shared between the two paralogs (Table 2; Table S10). The limited overlaps in DEGs and DAS events in the *smfa-1* and *smf-1b* mutants suggest that SMFA-1 is the predominant paralog acting in both gene expression and in splicing in Arabidopsis. These findings extend to the genome-wide level the functional non-equivalence that was observed with the two *smf* paralogs in the *GFP* splicing reporter system. Double *smfa-1 smfb-1* mutants are viable, and information about DAS and DEG events in the double mutant, which are essentially a sum of the individual *smfa-1* and *smfb-1* results, is shown in Tables S11 and S12, respectively.

## Discussion

In a forward genetic screen designed to identify factors involved in alternative splicing of a *GFP* reporter gene in Arabidopsis, we recovered sixteen splicing-related proteins that are predicted to have a variety of roles in the spliceosomal cycle and snRNP biogenesis pathway (Table 1). Based on the functions of their orthologs in other organisms, some of the factors we identified are likely to be components of a particular snRNP (U1: PRP39A, RBM25, RBP45D; U5: PRP8A). Others are predicted to be associated with a specific spliceosomal complex (complex C: DGCR14), or to be required at a specific catalytic step of splicing (step 1: CWC16A; step 2: PRP18A). Several factors are potentially splicing regulators involved in catalyzing or targeting various post-translational modifications, including protein phosphorylation (PRP4KA, CDKG2) and ubiquitination (RTF2, SMU1). We also identified factors presumed to be important for snRNP biogenesis (SMFA, coilin); mRNA transport (SAC3A); and transcription elongation/histone methylation (IWS1). One factor, CBP80, is potentially involved in multiple steps including splicing in the nucleus, mRNA export, and snRNA maturation in the cytoplasm. The data we gathered have verified a role for a number of previously uncharacterized proteins in pre-mRNA splicing in plants and revealed novel morphological and developmental phenotypes conferred by specific mutations. Our work has also generated new information on genome-wide gene expression and alternative splicing profiles of endogenous genes in the respective mutants. The overall findings provide foundational knowledge that can underpin more in-depth investigations of the splicing-related proteins in the future.

In addition to its foundational aspects, our study is strengthened and expanded in scope by merging and comparing data from the complete collection of mutants. A combined analysis permits a broader understanding of alternative splicing that cannot be gleaned from the examination of single mutants alone. For example, an overview of the current data set already illustrates the extraordinary complexity of splicing regulation. Even though different mutations often have the same effect on *GFP* pre-mRNA splicing and *GFP* expression, there is little overlap in the population of endogenous genes exhibiting splicing defects in the respective mutants (Kanno *et al.*, 2017a; Kanno *et al.*, 2018b). Substantial overlap would be expected if the mutations were revealing distinct, common features of introns affected in both mutants. Therefore, it has not been possible to discern fixed rules that govern splicing from the extant data. The findings rather indicate that each intron represents a unique context for splicing to occur, such that the effects of specific mutations on the splicing outcome are largely unpredictable (Pleiss *et al.*, 2007). The application of various post-translational modifications to splicing factors, which is suggested by our identification of putative protein kinases and ubiquitination targeting factors in the screen, adds a further significant dimension to the regulation of alternative splicing that is only beginning to be understood (de la Fuente van Bentem *et al.*, 2006).

Another interesting point arising from the cumulative findings is that obvious developmental phenotypes are observed primarily with GFP-weak mutants (Table 1). Although the biological significance of these findings is still uncertain, they may reflect different developmental roles for proteins required for the splicing reactions to take place at all *vs.* proteins that influence splice site selection but do not affect the occurrence of splicing *per se*. The latter category may be more important in plants for modulating splicing patterns in response to stress conditions or other transitory signals that induce an adaptive response.

Unlike their counterparts in yeasts and metazoans, many genes encoding splicing factors in Arabidopsis and other higher plants are duplicated but the extent of functional redundancy or functional divergence has not been clear in most cases (Kalyna and Barta 2004; Koncz *et al.*, 2012). In our mutant collection, we found a number of cases in which paralogs have a non-redundant function in *GFP* pre-mRNA splicing (Kanno *et al.*, 2017b, 2018a, b: this study). Paralogs of genetically-identified factors that we tested directly and found to be non-redundant in the *GFP* reporter gene system include *PRP39B*, *PRP4KB*, *PRP18B*, *SMFB*, and *RBP45A*, *B* and *C*. Although a direct examination of *CWC16B* was not possible owing to the lack of a suitable knockout T-DNA insertion allele, we presume that *CWC16A* is the main form involved in splicing *GFP* pre-mRNA because our screen identified three alleles of *cwc16a* and none of *cwc16b*. Similarly, *SAC3A* and *PRP8A* were the only paralogs of the respective genes retrieved in the screen, suggesting a specific role for the *A* forms in the *GFP* splicing reporter system. (Kanno *et al.*, 2017b, 2018a, b: this study). Although null mutations in these factors may be lethal, weak mutations that affect *GFP* splicing and expression could conceivably have been identified in the screen. For example, weak alleles of *rtf2* and *prp8* were recovered in the screen even though null mutations in these factors are embryo-lethal (Sasaki *et al.*, 2015; Kanno *et al.*, 2017a). Analysis of the RNA-seq data for both *A* and *B* forms of *SMF* revealed only modest overlaps in DEGs and DAS events between the paralogs, extending the functional non-equivalence of SMFA and SMFB that was observed with the *GFP* reporter gene to the genome-wide level. Extensive functional divergence of paralogous plant genes encoding splicing factors increases the complexity and capabilities of the splicing machinery and is likely to be another feature of splicing in plants that contributes to their developmental plasticity and ability to adjust to a constantly changing environment.

Another noteworthy aspect emerging from the combined analysis is that mutations were retrieved in splicing factors predicted to act throughout the splicesomal cycle and snRNP maturation pathways and not just at a single crucial step of *GFP* pre-mRNA splicing. The only possible bias concerns predicted constituents of the U1 snRNP, which is involved in recognizing the 5′ splice site early in the spliceosomal cycle. Of these, we identified three putative U1 snRNA components in the screen: RBP45D, PRP39A and RBM25 (Figure 2). The otherwise widespread distribution of the identified mutations as well as the participation of only single members of paralogous gene pairs at different steps of the splicing process hint that our system is illuminating a specialized pathway comprising a set of dedicated components.

The features of the alternatively-spliced *GFP* reporter gene that permit the genetic identification of a coherent set of splicing factors that cooperate in splicing of *GFP* pre-mRNA are not known. Potentially, however, they could be a subject for future investigation using CRISPR-Cas9-mediated genome editing to alter nucleotides within the spliced regions of the *GFP* gene. To our knowledge, many of the splicing-related proteins we identified have not been picked up in any other forward screen focusing on any type of process in plants, which again points toward one or more distinctive but as yet unidentified features of our *GFP* splicing reporter that render it particularly sensitive to mutations in the genetically identified factors. Based on the strong preference of their cognate mutants for the weaker AU-AC splice sites in *GFP* pre-mRNA, some HGF factors, such as CWC16A, SMU1, DGCR14 and CDKG2, might be important for discriminating between strong and sub-optimal/weak splice sites in the *GFP* pre-mRNA. Whether this observation extends to endogenous genes remains to be determined by scrutinizing in more detail the genome-wide RNA-seq data to assess both canonical and non-canonical splice site usage.

In summary, we used a unique alternative splicing system with both strong (GU-AG) and weak (AU-AC) splice sites, and an easy readout (*GFP* expression) to identify a coherent set of splicing factors that act at different stages of *GFP* pre-mRNA splicing and expression in Arabidopsis. The *GFP* pre-mRNA system is versatile and, similarly to other plant splicing reporters (Simpson *et al.*, 2012, 2014), can be used for other purposes, for example, to test mutations in other splicing factors or under different growth/environmental conditions. The resources generated in this study, including seeds of all mutants and high-throughput sequencing data, have been deposited in public repositories (Table 1) and hence are available to the international plant science community. We anticipate that additional global insights into the regulation of alternative splicing in plants will emerge once an entire data set containing detailed phenotypic and RNA-seq data from all mutants is available.
